# La Clinique des Hôpitaux des Enfans, &c. No. 10

**Published:** 1842-10-01

**Authors:** 


### La Clinique des Hopitaux des Enfans, etc. No. 10, January, 1842.

In this Number of the Clinique, the Editor has given an analysis of some excellent practical treatises on *tuberculous degenerescence*, as it occurs in children. The first of these is from the pen of Dr. Cheneau, under the following title, " may we not determine, at least to a certain extent, the causes of the predilection of tubercles for the lungs from the age of 15 to 40?"

During infancy and up to puberty, according to Dr. Cheneau, nature is anxious only about the preservation and growth of the individual. The life of this being, which is then altogether material, is concentrated in the organs of reparation and assimilation; the functional derangements of these organs must then favour still more the circumstances which preside over tubercular deposition, and accordingly tubercles are then more frequent in the mesenteric and intestinal glands. In support of his theory Dr. Cheneau states, that mesenteric and intestinal phthisis are more frequent than others, before puberty; this assertion, however, would require some proofs, more especially as it is obviously intended to serve as the basis of a theory. Until these proofs be adduced, we must withhold our concurrence; nay our own experience satisfies us of the falsity of the assertion, as we have reason to know that tubercles of the bronchial and pulmonary glands are more frequent than of the mesenteric and intestinal glands. Thus, " the abdomen being at this period of life the chief centre towards which Nature directs her efforts." Dr. Cheneau's theory explains, no doubt, the great frequency of abdominal tubercles at this age, but not their greater frequency than that of tubercles in other regions. The activity of organs is generally considered as the most powerful source of their predisposition with respect to tuberculisation, so that the organ most active is that most liable to be affected by its own activity. In this way the greater frequency of tubercles in the brain in the first years of existence is accounted for. M. Cheneau however denies the greater frequency of tubercles in the brain in infants, and explains this fact by the inactivity of the brain before puberty, the period at which the intellectual faculties first begin to be brought into play. And yet, according to our author, it is not the brain that will be the victim of its own activity, but the lung, on which organ the over-excitement produced in the brain by intellectual exertion and the various passions of the mind will be thrown through the medium of the pneumogastric nerve. Thus then, M. Cheneau, after having admitted in early age, the direct influence of the assimilating organs on themselves in producing tuberculization in the centre of their own activity, no longer admits it when the brain is in question, and yet, in order that his theory may be consistent, it is necessary that the cerebral excitability should produce on the brain itself and not upon the lung, that which the irritability of the digestive organs produces on these organs and not any other. " Life in infancy being," says M. Cheneau, " entirely material and limited to the abdominal functions, we may easily conceive that the disturbances of the nervous influx should be directed in preference to the organs contained in this cavity, and that the formation of tubercles should be more frequent there, whilst, once puberty is completed, and the brain now performs functions in which it participated heretofore but in a very slight degree, the continual disturbances thence resulting, must in their turn, modify the organs with which it holds *direct relations,* and thus pulmonary tuberculisation must take place in the lungs in preference to any other organ." To this, however, it may be objected that it is the brain itself which ought to become modified ; for there is no organ to which it is more *directly* related than to itself, and the disturbances of nervous influx should pass in preference to the brain, in the same manner as they do to the organs which are the seat of the abdominal functions. Another objection that may be advanced to this theory is that the brain is more directly connected, by the pneumogastric nerve, to the stomach than to the lung, so that it is to the former organ that the nerve should convey the influence of cerebral excitability and produce morbid changes in it.

Before we close our notice of this article, we beg leave to remark that both the author and his critic seem to us to understand the principle which regulates the morbid predisposition of certain organs at different periods of life differently from other people. M. Cheneau has it that the greater the activity of any organ is at any period of life, the greater is its liability to tubercular disease at such period. We always thought that the principle was, that the more developed any organ is at any period of life, compared with others, the greater is its liability to become the seat of disease. It is a fact which cannot be questioned, that young children are more liable to cerebral disease, than adults, and it is equally certain that the brain in them is more developed than any other organ of the body. The activity of the abdominal organs in discharging their several functions is entirely a different kind of thing from the activity of the brain in thinking—the difference is precisely the same as that between animal and organic life. We think that there is some misconception here with respect to the principle, which regulates morbid predisposition, and also an abuse of the term " activity."

The next essay on tuberculisation is by Dr. Becquerel. An analysis of it is contained in No. 11, of the Clinique, February, 1842 ; it is entitled *Bronchial Ganglionic Phthisis in Children.* We shall present our readers with the most interesting points in this essay.

Tuberculous degenerescence of the lymphatic glands which surround the bifurcation of the trachea and bronchi is observed more frequently than pulmonary tuberculisation. The tuberculous glands may be situated beneath or around the tracheal bifurcation, around the large bronchi, at the root of the lungs, or between these tubes.

*Anatomy.*—In the state of crudity there is hypertrophy of the glands. Either in the state of a grey semi-transparent substance, or, at a later period, in the state of crude tubercle, the glands present themselves distinct and separate, or several of them soldered together ; they sometimes acquire the size of a pigeon's egg. When soldered together, they may form a mass which surrounds the large bronchi and the great vessels situate at the base of the neck.

*Destruction.*—Under the influence of enlarged glands, there may be destruction of the bronchial parietes, or at all events compression without destruction of the parietes, and a diminution in the calibre of the bronchus.

*Compression.*—The pulmonary artery and pulmonary veins, the aorta and the venæ cavæ, after their exit from the pericardium and a little below the base of the heart, are sometimes compressed; the pulmonary artery and pulmonary veins more frequently, sometimes the aorta, and hence hypertrophy of the left ventricle of the heart. The venæ cavæ, superior and inferior, are sometimes compressed simultaneously, sometimes separately. Attention to this compression is solicited by M. Becquerel, because under certain circumstances, it accounts satisfactorily for certain hypertrophies which become developed in phthisical subjects.

Tuberculous and enlarged glands may project anteriorly, may become adherent and soldered as it were to the sternum and more especially to its upper part.

*Pus.*—In a paragraph which treats of softening of tuberculous bronchial glands, Dr. Becquerel cites the case of a child five years old, in whom he met five or six bronchial glands filled with good pus without any lumps. On making an incision into them, he says, we observed a cyst lined with a membrane injected so as to present the appearance of the branches of a tree, without any remnant of tubercles; and as there existed no tubercle nor gland either in the lungs or in any other organ, one might have admitted a non-tuberculous suppurative inflammation of the glands, if M. Becquerel had not found, by an attentive examination, three small bronchial glands presenting all the appearance of the grey induration, and two others transformed into crude tubercles.

Ganglionic tubercles sometimes undergo a modification of a suet, steatomatous appearance. This modification may be either indicative of commencing softening, or else of some change tending to a cure, or the transition to the cretaceous state; the latter state is very unusual in children.

*Cavities.*—Tuberculous matter, once softened, must be discharged sooner or later. This mode of evacuation of these cavities takes place through the bronchi, the parietes of which become ulcerated from without inwards and give exit to the softened matter. The ganglionic cyst communicating with the tube constitutes a *false cavity.* When these false cavities replace inter-bronchial glands somewhat deep-seated, they are easily mistaken for true cavities.

These cysts, when they are multilocular, may go on to a certain depth, perforate the pulmonary pleura, and produce hydro-pneumothorax. They have been twice found to produce ulceration of the pulmonary artery, and to occasion perforation of the other blood-vessels, as also that of the œsophagus.

Cysts, formed by the membrane of the glands, which become cicatrised by adhesion of the parietes, constitute another mode of termination of ganglionic phthisis.

Tuberculisation of the bronchial glands may exist alone and distinct, or it may coincide with a similar degenerescence in the mesenteric glands, with tuberculous ulceration of the intestines, or with tubercles developed in other points of the system. Sometimes it is observed to coincide with tuberculous degenerescence of the lymphatic glands of the neck.

*Causes.*—The causes of this disease are but little known. In infancy the tuberculous affection may assume, according to M. Becquerel, four distinct forms:

1st. The different lymphatic glands of the neck, chest, and abdomen undergo tuberculous degenerescence, separately or simultaneously.

2. The tubercles are developed in the different parenchymatous structures: in the lungs, brain, liver, spleen, and kidneys.

3. They are developed simultaneously in most of the organs, under the form of what are called miliary granulations.

4. They are more especially developed in the sub-serous tissue of the different cavities. They almost always occasion a chronic inflammation of the serous membranes with which they are in contact.

Ulcerations of the intestines may shew themselves under all forms, either distinct, or combined.

The causes of tuberculisation in children are the following: hereditary taint, being born of parents aged or unhealthy; delicate, lymphatic constitution of parents; bad nursing; unwholesome, non-nutritious diet; dwelling in a cold, moist, and ill-ventilated habitation; harsh treatment; exposure to cold by insufficient clothing; masturbation; convalescence from measles, scarlatina and typhoid fever; any disease of long duration by weakening the system.

*Diagnosis.*—The author justly observes that the symptoms of tuberculisation are so obscure, that it is extremely difficult to diagnose them. The new results which he gives belong to a considerably advanced stage of the disease.

Sometimes *pain in the region of the sternum.* On a line with the sternum there may be a feeling of dull pain, which patients generally refer to the stomach. It is sometimes observed in children, in acute or chronic bronchitis, and in the different forms of tuberculisation.

*Cough.*—In all pulmonary affections.

*Dyspnœa.*—When tubercular and very much enlarged glands compress the trachea at its bifurcation or near the large bronchi, this is very intense. Considerable dyspnœa is characteristic of this affection, when it cannot be accounted for by the phenomena of auscultation, which if they are not entirely absent, indicate the existence of other affections which, for their extent, would be far from being sufficient to account for the difficult respiration experienced by the patient.

*Expectoration.*—When there is enucleation of the degenerate gland, and this has made its exit through an opening in the bronchi, it may serve for a diagnosis.

*Percussion.*—This cannot be useful unless the diseased glands have penetrated to a certain depth of the lungs, or to the surface.

*Auscultation.*—Compression diminishing the calibre of a bronchus, which very rarely happens, the result may be a diminution in the intensity of the respiratory murmur in the part of the pulmonary tissue to which the bronchus is distributed.

We may admit as rational, the possibility of the following symptoms, though they may not have been observed: if a considerable number of tuberculous glands approach the surface of the organ, there will be a murmur of *prolonged expiration ;* if these glands are converted into cysts communicating with the bronchi, there will be *gurgling ;* if the pleura be perforated, there will be hydro-pneumo-thorax.

Sometimes *palpitations,* when the large vessels are compressed by large glands. This chiefly happens, when the latter compress the aorta.

Tolerably often dropsies, and more especially greater or less œdema of the lower extremities and of the trunk, when there is compression of the vena cava, and especially of the vena cava inferior. In phthisical children dropsies are somewhat frequent. When anasarca comes on at an advanced stage of the disease, we may be well-nigh certain that the serous infiltration is owing to a cachectic state of the system. At a less advanced period, we may fairly suppose that it is the consequence of compression of the venæ cavæ, more especially of the inferior, a circumstance which causes the infiltration of serum to occupy most frequently the trunk and lower extremities.

The examination of the urine may discover Bright's disease, which sometimes complicates tuberculisation in children.

Tuberculisation of the bronchial glands, when it attains the stage of suppuration, may give rise to the following general symptoms: loss of strength, emaciation, hectic fever, especially in the evening and at night; sometimes diarrhœa, most frequently connected with intestinal ulceration. We may add the frequent symptoms of complication of tubercles, either of the lymphatic glands of the neck, or of the lung, or of the mesentery and peritoneum, &c.

All the symptoms now described may be completely absent; this happens at the first stage of the disease. They may be slight and but little marked; and without an attentive and minute examination they may escape the observer. They may be masked by the symptoms occasioned by other tubercular affections, as of the lung, for instance; a child presents at the apex of the two lungs softened tubercles and cavities; he coughs, becomes emaciated, has dyspnœa, &c., and auscultation detects at the same time the change produced in the lung; we will naturally attribute to this lesion all the share in the phenomena observed. If tuberculisation takes place on the part of the cervical glands, we shall be deprived of some symptoms which are common: emaciation, hectic fever, and also dropsies, if tuberculisation exists in the abdominal cavity; we are then no longer warranted in attributing these symptoms to bronchial tuberculisation.

Mode of *termination* of the disease:—rarely a cure by a resolution of the tubercles which is supposed rather than proved; 2, an enucleation and discharge of the tubercles by cough; 3, conversion of the tubercles into cretaceous matter; 4, softening of the gland and its opening into the bronchi, expulsion of the tuberculous matter and cicatrisation of the cyst. This termination which is possible, is not proved anatomically.

*Frequent Death:* 1st, by the progress of degenerescence; 2, by the development of an accidental and acute inflammatory complication, bronchitis, pneumonia resulting from the debilitating influence of this degenerescence, especially when it occasions suppurating cavities; 3, by the development of other lesions of a tuberculous nature; tubercles of the lungs, pleuræ, tubercular peritonitis, tubercular ulceration of the intestines, tubercular meningitis.

*Prognosis* almost always very bad. This disease, essentially chronic as it is, is marked by remissions and exacerbations.

*Treatment:* the same as that of tubercular disease in general; recourse must be had to suitable hygienic means, to tonics and strengthening means.

When the local symptoms assume a certain degree of intensity, particular means must be resorted to: thus when the dyspnœa becomes urgent, local bleeding and blisters; when there is dropsy, dry frictions, and diuretics; purgatives should be avoided, as they might occasion a diarrhœa difficult of being arrested. Any accidental complications must be combatted: diarrhœa by astringents united to opiates; local inflammations by blisters rather than bloodletting, which would reduce the system too much.

The last of these articles is from M. Berton on Bronchial Phthisis. (No. 15—April, 1842.)

*Pathological Anatomy.*—Alterations of the lymphatic glands of the bronchi predispose to inflammation of the respiratory organs and favour the development of tubercles of the lung: witness, says M. Berton, the tenacity, tedious course, and the returns of inflammation of the chest and the frequency of pulmonary tubercles in children. And if these alterations of the bronchial glands predispose the organs of respiration to inflammations, it is equally true that, under the influence of inflammation of a neighbouring organ, the alteration of the bronchial glands makes rapid progress.

What assigns so important a place to ganglionic phthisis in the pathology of tubercular affections is the organic lesions which the presence of enlarged glands may occasion in the neighbouring parts. Though bronchial tubercles may acquire considerable size without producing displacement, yet an extraordinary development of some of these organs displaces the neighbouring parts when they are moveable. M. Berton twice found the œsophagus pushed to the right.

Perforations take place at the point of contact of the neighbouring parts with the diseased glands; consequently the ulcerative inflammations which produce them manifest themselves towards the period at which the tubercular matter softens, unless the glands have a tendency to compress the surrounding organs, for then the adhesions and perforations already take place, whilst the tubercles are still in the crude state. These two occurrences were observed in two cases cited by M. Berton. In one, the inflammation of the cyst of the gland, a more intimate adhesion to the neighbouring branches, ulceration and perforation followed the softening of the tubercular matter. In the second, the tubercle was still in the crude state, and its situation explained how, from its tendency to compress the neighbouring organ, an adhesion took place, subsequently ulceration, and then perforation of the compressed organ.

The existence of ganglionic cysts which have passed into the osseous or cartilaginous state, containing cretaceous matter, a thing frequently observed, leads one to think that the softened tubercular matter may sometimes be absorbed either in part or entirely. But the most usual tendency is to be discharged by perforations, which are established chiefly on the bronchi.

One cannot help feeling astonished that enlarged bronchial glands, observed by M. Berton as capable of producing perforations in the bronchi, should be considered by him as incapable of compressing these organs. He explains it in this way ; these bronchial glands perforate the bronchi by the process of ulceration occasioned by their contact, so that we may conceive this process set up even before any considerable compression can take place.

*Causes.*—The bronchial glands may become affected, according to M. Berton, when the morbific causes act on the system to which they belong, or on the organs with whose functions they are more immediately associated. Thus, of the four forms which the tubercular affection may assume, according to M. Becquerel, (in the preceding article,) whatever be that which the causes occasion in a tuberculous individual, the bronchial glands may at the same time feel their influence.

Inflammation of these glands is very uncommon, according to Laennec : this proposition is true only with respect to adults ; for in the early period of life all the causes capable of developing in an inordinate degree the irritability of the glands and of the lymphatic vessels in general, and of the bronchial and pulmonary glands in particular, dispose them to inflammation and tuberculisation ; so that tubercular degenerescence of these glands is a frequent result of their inflammation. The bronchial glands soon become inflamed when an irritating cause has directed its action to an organ, from which lymphatic vessels proceed which are functionally related to them.

The author here generalises the influence of inflammation of the neighbouring organs on the production of tubercular degenerescence of the glands. Thus that of the gums accompanying dentition, and ophthalmia and affections of the hairy scalp, occasion swelling, and tuberculisation of the glands of the neck ; that of the intestinal organs of a certain duration produces tuberculisation of the mesenteric glands in young subjects (tabes) ; in the same way, under the influence of pneumonia and prolonged bronchitis in children, the bronchial glands become inflamed and transformed into tubercles.

The inflammatory origin of such a transformation, says Dr. Berton, appears more particularly evident in *bronchial phthisis.* It is sometimes possible, says the author, to surprise, as it were, the cause producing the effect, to detect in fact the *tuberculous degenerescence* coming on after an inflammatory process. On examining certain bronchial glands, we find evident traces of inflammation and the production of tuberculous matter differently united and combined. We shall not enter here with the author into the details of pathological anatomy; what we have just said on this subject will suffice to shew what the author's opinion is regarding the power of irritation and inflammation as a cause of tuberculisation. His opinion is founded on the anatomical examination of the bronchial glands, partly inflamed and partly tuberculated.

Morbid change of the bronchial glands presented itself to M. Berton's researches under three different forms, and what proves inflammation to be an invariable cause of these varied effects, is the constant coincidence of the following pathological states: pneumonia, bronchitis, pleuritis, phthisis. The three forms of alteration in the bronchial glands were:—1st, red, enlarged glands; 2d, glands partly inflamed, and partly replaced by tubercular matter; 3d, glands entirely tuberculated.

Even when inflammation of a viscus or of its appendages may not have preceded the inflammation of the lymphatic glands associated in function with this organic apparatus, still inflammation was to be complained of. The irritability of the lymphatic system may increase primarily and essentially (as in the lymphatic or scrofulous temperament); excitement brings on irritation; the latter induces inflammation and subsequently *tuberculisation*. Thus we may conceive the extension of *scrofulous disease* developing pulmonary tubercles in individuals who have never coughed.

Might not, asks M. Berton, the granulations described by Bayle within the pulmonary tissue, be pulmonary lymphatic glands swollen, enlarged and so rendered visible? The existence of these greyish bodies of the size of a millet grain, frequently coincides with that of scrofulous affections: according to Baron, they are often observed in weak and rickety children. Like the scrofulous disease itself, they are seldom observed in infancy before the first dentition.

The question of the influence of inflammation on the production of tubercles is important with respect to treatment.

It is in some degree the immediate cause of this affection. Thus the physician should treat in the most decided manner pulmonary, bronchial and pleural inflammations in children. The danger of imperfect resolution is to be particularly dreaded at this period of life. With respect to the remote causes, they are to be considered under the head of hygiene. In the first rank are to be considered all those causes which are capable of developing the *scrofulous taint;* such as damp habitation, bad aliment, deprivation of pure air and of light, &c.

Bronchial phthisis is very common in children of a lymphatic constitution during the period comprised between the two dentitions. The greater activity of the lymphatic system in early age, explains this general fatal predisposition of the glands in children.

*Symptoms.*—The *symptoms* of bronchial phthisis have been described according to the ideas suggested by the knowlege of the organs connected with the enlarged bronchial glands and the phenomena which should result from the compression of these organs: impediment occasioned in the circulation of the blood, hypertrophy of the heart: obstruction of the œsophagus, trachea and bronchi, &c.

An attentive examination of facts induces M. Berton to deny the truth of all these anticipated symptoms just enumerated. We shall present the reader with a rapid glance at the author's remarks. He gives seven cases of bronchial glands enlarged and tuberculated.

In the first no remarkable organic lesion was produced by their presence: in the second and third, there was perforation of a branch of the bronchi; in the fourth and fifth, there was perforation of one bronchus and of the œsophagus; in the sixth and seventh, there was perforation of the pulmonary artery.

Out of these seven cases of bronchial glandular phthisis, paleness and emaciation were noticed only twice; in only one of these patients was there observed a good share of embonpoint; œdema was noticed only once; in three the glands of the neck were engorged; in two they were not so. In all there was some cough of several months' standing. In two the cough was frequent at the time of the examination. No expectoration in any. Embarrassed respiration and great oppression were noticed only twice; in one of these two cases the respiration was abdominal. Sonorous rhonchus once; crepitating rhonchus twice; mucous rhonchus twice. Pulmonary expansion less free than natural in two instances.

In two of these cases percussion and auscultation detected nothing. In those cases in which percussion and auscultation were negative, the trachea, larynx and bronchi were pale, except in two, where the bronchi were found somewhat red. In the contrary cases the trachea, larynx and bronchi were injected; the bronchi contained puriform mucus; there was adhesion of the two pleuræ and of the lobes of the lung; there was observed partial hepatization, pulmonary tubercles, and granulation.

Only once the pulsations of the heart were observed to be extended, and at the autopsy this organ was found to be enlarged. Thrice the state of the skin was noted, and it was set down, as hot, cold, and of natural heat. The pulse which was always regular was observed to be in four patients, 76, 96, 110, 120. In this last case there was some fever. The pulse marked 76 belonged to the child whose heart was found to be enlarged. Great debility in two of these children, one of whom had a frequent, deep cough, and the other diarrhœa; headache in one of those that had fever. There was vomiting in two children, who at the same time had diarrhœa, and both were found to have a perforation of the œsophagus communicating with one of the bronchi; one of these two had gangrenous stomatitis. In two cases only was hæmoptysis observed which proved rapidly fatal; in these perforation of the pulmonary artery was observed after death. In one of the two cases where there was fatal hæmoptysis, clots of blood were found in different parts of the air-tubes. Only once M. Berton found clots of blood in the heart; this was the case wherein he discovered no perforation at the autopsy, and in which there had been serous effusion, and abdominal respiration. The aspect of this case is different from those in which ulcerative perforation took place. One would be tempted to consider this difference as the effect of greater compression in this case; but the author, after having said that large tubercles surrounded the origin of the large vessels, adds that no real compression resulted from them.

The bronchial glands, when tuberculated and enlarged, cannot, he says in another place, compress the bronchial tubes so as to flatten them, a compression which is not announced by any peculiar phenomenon, such as a particular sifflement, jerking respiration, &c. So that the dilatations of the extremities of the bronchial tubes, which are tolerably frequent in children, should not be attributed to this compression.

The new adhesions of diseased bronchial glands to the neighbouring organs, as also the perforations occurring at the points of contact of these organs with the bronchi, give rise to no particular symptom. With respect to the sternal pain, it belongs, according to M. Berton, to the symptomatology of bronchitis.

To suppose that in cases where a communication is established between the tubercle and the perforated bronchus, the absence *of pectoriloquy*, and the expectoration of fragments of tubercular matter, will suffice to throw light on the diagnosis, would be to expose ourselves to serious mistakes. In one of the cases, which was an instance of perforation of a bronchus, there was no expectoration of the tubercular matter, which was softened; the opening of the bronchus was plugged up by a lump of this matter contained in the glandular cyst. Besides children expectorate but little. With respect to pectoriloquy, it is very uncommon in children. According to M. Berton vomiting is not a symptom which can throw light on the diagnosis; for besides that, on the one hand, children have a great disposition to vomit during the progress of all their chest affections; on the other hand, it happens that in the cases of accidental communication established between a bronchus and the œsophagus, simultaneously perforated by the purulent abscess and the discharge of the enlarged glandular substance, it happens that, even in these cases, neither vomiting nor coughing takes place, though one might be warranted in supposing, that some portions of the fluids swallowed pass from the œsophagus into the branches of the bronchi. M. Berton has observed children who, under such circumstances, had not coughed after drinking.

It would appear that the author, in his appreciation of the facts, has not sufficiently distinguished from each other the phenomena of coughing and vomiting; and that which is applicable to the one, even according to M. Berton's observations, is not applicable to the other. Accordingly, in the seven cases, which have just been sketched, there was some cough, whilst, in only two of them vomiting took place, and these two patients are precisely those who presented at the autopsy a corresponding opening of communication on the œsophagus and on a bronchus, between which a diseased glandular cyst was found.

Thus, in one of M. Berton's cases, where there was a communication established between the œsophagus and a bronchus by the simultaneous perforation of these two canals, M. Berton says, that a period prior to his admission into hospital, this child had been ill, that he had coughed and vomited. This circumstance, combined with the fact that the edges of the perforations presented no trace of recent inflammation, might induce one to think, as the author himself says, that the vomiting had coincided with the establishment of the communication between the two organs.

In another case there was the same accidental communication established between the right bronchus and the œsophagus, by the presence of a softened tuberculated gland, and in this case again there was vomiting in the little patient; this vomiting supervened at the time when the countenance became entirely changed, fever increased and diarrhœa set in. Here we may remark that the vomiting had been observed by M. Berton only in those very two cases, where the autopsy detected a perforation of the œsophagus corresponding to the opening of a bronchus in connexion with a softened gland, a pathological state capable of causing the phenomenon of vomiting; just as the hæmoptysis, promptly fatal in the one case, and very severe in the other, was observed only in the two cases, where perforation of the pulmonary artery was discovered at the postmortem.

In another of M. Berton's cases there was perforation of the pulmonary artery: measles had occurred at the age of eighteen months; the cervical glands were engorged; admitted into hospital at the age of three years and a half: enteritis, bronchitis; pulse feverish, the abdominal symptoms engrossed all the attention of the physician. Suddenly there occurred severe hæmoptysis by the mouth and nose. At the autopsy some clots of blood were observed in different parts of the air-tubes of the left side. An immense cavity communicated with a bronchial cyst, which itself communicated with the left trunk of the perforated pulmonary artery. There was not a single phenomenon which could cause a suspicion of the morbid process, which was secretly preparing the fatal event.

Must we invariably despair of a cure in cases like this? may we not conceive it possible, that after the evacuation of the softened tuberculous matter, the cyst and fistulous openings may cicatrize? Such cases, M. Berton says, can only be considered as partial, and not definitive. In our analysis of M. Becquerel's essay we have already had an opportunity of observing that this mode of termination was not proved anatomically. Besides, adds M. Berton, when the bronchial glands have become tuberculated, it often happens that tubercles are developed at the same time in the lungs.

The author, by way of recapitulating the elements of the diagnosis, reduces them to the following data: the morbid state of the bronchial glands can only be suspected. Our suspicions acquire probability, if the patient be of a lymphatic temperament; if he carries about with him some marks of the scrofulous taint; if he has coughed for a considerable time; if for the last two or three months he is affected with bronchitis, peumonia, &c.; scarcely, are there any rational signs, he adds, which can serve to throw light on the diagnosis of bronchial phthisis. The author of the analysis of M. Berton's essay well observes here, that M. Berton does not appear to take sufficiently into account all the aid that may be derived from the comparative method of diagnosis. Let us take, for example, the phenomenon of dyspnœa: if this phenomenon cannot be accounted for in a patient by any other affection, whether this affection does not exist, or whether, if it does, it is not sufficient, however, to produce the great oppression observed; if, moreover, there exists a sternal pain without severe bronchitis, we shall have the elements of an almost certain diagnosis. Let us add the several phenomena which might result from compression of a bronchus (diminished intensity of the respiratory murmur) from compression of the surface of the lungs (prolonged expiratory murmur, &c.), from compression of the large vessels (palpitations), from compression of the vena cava (dropsy, œdema); a group of phenomena indicative of hypertrophy of the bronchial glands, at a time when it could not be accounted for by any other lesion.

Thus to the direct signs of presumption enumerated by M. Berton, we should add the indirect signs arising from the absence of the symptoms, which would characterise another affection equally suspected, that is, we must establish the diagnosis by the comparative method and by the process of elimination.

### ETUDES SUR LES RAPPORTS DE LA GROSSESSE ET DE L'ACCOUCHEMENT AVEC L'HYGIENE DE L'ENFANT AVANT ET APRES SA NAISSANCE.

*Observations on the Relations subsisting between Pregnancy and Delivery, and the Health of the Infant before and after Birth.*

The first question which presents itself to us with respect to all that concerns the health of the fœtus is, the influence exercised on it by the physical and moral conditions of the mother. These impressions may be felt by the infant even before birth. The importance of addressing to pregnant women, useful instruction which may inspire them with correct ideas regarding their new state, and which may withdraw them as well as their infant from the mischievous effects of the prejudices which beset them at the very time when they are most liable to impressions, cannot be questioned.

*Diseases of Pregnant Women.*—Under this head may be included various physiological states, such as eccentric tastes, irregular appetites, &c., which are observed to occur in pregnant women, and which, though they are often but mere annoyances, are still capable of disturbing the functions, and may become dangerous by being allowed to proceed. We shall here describe the effects which these different circumstances may produce on the fœtus, and the means of preventing either the diseases themselves, or their influence on the child.

Pregnancy being a natural state produces no disease of itself; it is only the occasion of the development of some affections which depend on certain dispositions of the economy. Acute diseases; fevers, inflammations, eruptive fevers, and certain chronic affections, produce mischievous effects on the fœtus, the former by their violence, the second by their long continuance. Galen long since remarked that inflammatory diseases cause the fœtus to perish. The child may escape this danger; but then, either the mother, in order to ward off death, subjects herself to a strict regimen, and in this case the child dies for want of nourishment; or else, for the purpose of preserving the life of the child, she takes food, and then the disease becomes more severe, and if the mother is not carried off by it, the infant at least perishes. When, in pregnant women chronic affections come on: intermittent fevers, cough, jaundice, spasmodic and cachectic affections, they are so much the more injurious to the fœtus in proportion as they are intense, recent, contagious, as venereal diseases, small-pox, measles, &c.

Such are the sympathies which subsist between the mother and child, that, according to some authors, circumstances occurring in either affect both. However there are certain morbid dispositions peculiar to the fœtus and in which the mother does not participate.

To the division of pregnancy into three periods, each consisting of three months, correspond three groups of symptoms, the first of which includes the following phenomena: nausea, vomiting, disrelish for food, depraved, inordinate appetite, cardialgia, pains in different parts of the body, hiccup, vertigo, lassitude, oppression, diarrhœa, loss of blood, cachectic states of the system, weaknesses, syncope, &c. The group corresponding to the second period consists of the following phenomena: cough, palpitations, acidity of stomach, weaknesses, hæmorrhages, relaxation of the uterus, loss of sleep, pains in the loins and thighs, &c. The group of phenomena accompanying the third period consists of suppression or incontinence of urine, difficulty in passing urine, constipation, tenesmus, hæmorrhoids, varices in the legs and thighs, swelling and œdema of the lower extremities, serous discharges through the vagina, spasms of the uterus, peculiar disposition to fall, &c.

All these circumstances, even those which appear least severe, disturb the system, and give rise to diseases dangerous to the mother and child. It is necessary then to be aware of the morbid effects resulting from them in order to be able to prevent them. It must be kept in view that these diseases are not pregnancy itself, but that they take their source in certain general and peculiar causes with which it is important to be acquainted.

To the cessation of the menstrual discharge may be attributed the diseases which come on in the first period of pregnancy; those which come on at other times, to dilatation of the uterus, to its increase in size, and to the compression made by it on the viscera, muscles and vessels.

If women who conceive found themselves at the time in all the conditions of perfect health, they would not be exposed to that long train of annoyances, which distress them during the entire period of gestation. Women who enjoy good health, who take moderate exercise, and adopt a judicious regimen, and are not subjected to painful moral influences, are not exposed to such distressing annoyances, nor their infant to the morbid affections which are the constant consequences of them. If all the organs were perfectly free, and were able to discharge their functions without constraint, if there was not in certain parts of the system an excess of debility and irritability; if the blood, which, before pregnancy, discharged itself periodically only because it was abundant and superfluous, could circulate freely during pregnancy when it becomes necessary; if, in one word, the causes which produce illness and the affections connected with the first period did not exist, we should witness less of the diseases of the two other periods, which are connected with each other, as also with those of the first period; we should not see those cachectic conditions of the system, which compromise the lives both of the mother and the child, produced by those causes, the principal of which is the inequality of the circulation.

Conception produces in women a kind of general shock, which affects all the senses. In delicate women this shock leaves after it a disposition which may lead to a degeneration of their functions. The new being formed in the uterus of the mother, requires new directions in the distribution of the liquids and the movements of the solids. If the woman possess within her resources sufficient to maintain the play of the new functions, her health will hold up. Let this order of functions appropriated to the wants of the mother and the fœtus happen to become deficient, then annoyances and diseases are sure to come on, which will direct their action to the mother and the fœtus. Thus, in a delicate woman, the periodical discharge of the menstrual flux follows its natural course; she becomes pregnant; on a sudden pregnancy gives entirely new directions to the phenomena of the economy: an irregular distribution of blood in the vessels, an irregular distribution of all the liquids, disorder in the movements of the solids, disturbance in the functions; hence arise, in such a woman, the principal diseases of pregnancy.

If the cessation of the menses were the real cause of the affections to which pregnant women are liable in the first period of pregnancy, robust women would not be more exempt from them than delicate women.

What are the causes of the eccentric and irregular cravings, which make them covet highly-seasoned meats, salted and spiced food, and spirituous liquors? Is the cause of this affection a certain weakness of the stomach, which the woman instinctively seeks to relieve by means of food capable of stimulating the digestive organs? Does the impaired or the depraved appetite arise from the circumstance, that in consequence of the imperfect digestion, the juices necessary to this process become changed, as the saliva and gastric juice; or from this, that the bile, badly elaborated, becomes depraved, and from the mixture of these liquids in the stomach, irritations are established in this organ, which blunt and pervert the taste? What we have said of the bile is not a mere theory; it is a fact demonstrated by the irregular excretion of fæcal matters which have a cadaveric odour and a greyish colour.

The cardialgia is a violent pain felt more particularly at the upper orifice of the stomach, and which equally affects the pylorus or lower orifice of the stomach, as well as the rest of this viscus. It is accompanied by swooning, palpitations, cold sweats, great restlessness, oppression, depression of strength, desire to vomit, spasmodic shivering of the extremities, some general nervous movements, &c. What are the causes of this pain?

1. It is frequently spasmodic and windy.

2. It sometimes depends on the presence of irritating liquids in the stomach and duodenum. This latter kind of cardialgia may be distinguished from the others by the few symptoms which accompany it; it occurs only after a series of bad digestions, whilst spasmodic cardialgia frequently comes on soon after conception.

3. Among the causes of violent cardialgia mentioned in books, we may cite the presence of gravel retained in the urinary passages, and of stones impacted in the bile-ducts.

4. The presence of corrosive poisons taken into the stomach, and of emetics and drastic purgatives.

5. The sudden suppression of an epidemic dysentery.

6. Mental annoyance and violent passion, by the violent impressions which they make on the nervous system.

7. In pregnant women the presence of a certain quantity of blood retained in the membranes of the colon and rectum, carried thence by metastasis into those of the stomach, chiefly towards the cardiac orifice. Such metastases, if they took place, would soon be followed by fever and inflammation, and then other symptoms would manifest themselves besides those of cardialgia.

8. The copious afflux of blood towards the stomach and diaphragm; but we may say that this is but an inference from what has been ascertained by post-mortem examinations in the case of persons who have died of *stomach* spasmodic asthma, and in whom the obstructions to the circulation, and the consequent afflux of blood to the stomach and diaphragm were accounted for by the presence of polypous concretions in the heart.

9. The suppression of hæmorrhoids, the suppression or retardation of the menses, or rather the cause which suppresses or suspends them. Accordingly we often find that in a pregnant woman plethora occasions cardialgia every month, at the time when the menses used to appear before pregnancy. Besides the cardialgia the pregnant woman may feel pains in the region of the kidneys, in the loins, in the breasts, head and teeth. To this may be added the great heaviness of the entire body, lassitude of the limbs, symptoms which all arise from the same sources; delicacy, weakness, irritability, erethism of the tissues or violent tension of the fibres. All these pains may be rendered still more acute by dilatation of the uterus, extension of its ligaments, and the weight of this organ on the painful parts. To the broad ligaments may be referred the pain of the loins, to the round ligaments those of the groins, pubis and thighs.

Erethism of the parts of the pelvis impedes the course of the blood towards the lower extremities; this fluid flows back towards the upper parts; it flows but tardily, and is distributed irregularly. This occasions vertigo and pains in the head, as also an afflux of blood towards the spongy substance of the gums, and then pains in the teeth, slowness and irregularity of the circulation of the blood in the lungs; this gives rise to great oppression and palpitations; then the pulse becomes weak and languid.

When the uterus is suffering, the breasts are ordinarily affected at the same time. As the circulation does not go on freely in them, they swell and become painful; the same thing happens to several women at the approach of menstruation. In plethoric women the retardation of blood in the vessels occasions engorgements, which encrease the symptoms attributable to the delicacy of the tissues, to their state of relaxation, as also to their erethism and irritability, phenomena which have themselves a cause independent of pregnancy, and which are always occasioned by abuses committed in diet.

Hiccup, convulsive contraction of the œsophagus and diaphragm with sudden and sonorous inspiration, is sometimes so constant and so violent that we have seen women actually menaced with suffocation from them. The state of pregnancy being a predisposing cause of this painful convulsion, pregnant women should know how to withdraw themselves from the causes which may induce it. We shall explain these, when we come to treat of the means of preventing the affections which may come on during the first period of pregnancy.

Pregnant women are often affected with dizziness occasioned by the derangements which occur in the functions of the stomach, and by disturbance of the other organs of digestion. All objects appear to them to revolve around them, and they themselves seem to themselves to turn round. Sometimes this dizziness is announced by precursory signs : heavy dull pain of head, tinnitus aurium, and vomiting. The explanation of this dizziness has been sought for in the pressure made on the nervous plexuses, in the irritation occasioned by the contact of the depraved products of secretion, such as the bile, and in the effects produced by bad digestion. It is well known that, by the effects of nervous communications or sympathies, the stomach suffers when the head suffers, and that the head becomes soon affected, when the functions of the digestive organs are deranged. These organic sympathies are frequent, more especially in pregnant women of a delicate constitution.

The intestinal fluxes observed during pregnancy are, diarrhœa, frequent discharge of liquid, bilious, serous, and sometimes even purulent matters; dysentery, with frequent and bloody evacuations, accompanied by cutting pains which traverse the entire intestine, universal distaste for solid and liquid aliments, and sometimes by febrile re-action ; sometimes there is lientery, characterized by frequent fæcal discharges. In these cases such is the state of irritation, and consequently the state of sensibility of the intestine, that it cannot tolerate the presence of alimentary substances, which are discharged half-digested. Another of the annoyances of pregnancy is tenesmus. This state is often a consequence of dysentery, and of inflammation of the rectum ; it is sometimes attributable to bilious, acrid humours, which are contained in this intestine, and irritate it: perhaps, in some cases it is owing to the presence of ascarides, or of some tumor around the rectum, of gravel lodged in the neck of the bladder, &c.

It sometimes, though rarely, happens that some women continue to have the menstrual discharge during the commencement of pregnancy, sometimes even to the sixth month.

The cachexia which occurs in some pregnant women, is characterized by general disturbance of the functions, paleness and general softness of the skin ; in a word, debility, languor, extreme depression, restlessness in the limbs, difficult respiration, stifling and syncope; during the syncope there is cold sweat, pulse imperceptible, loss of consciousness, sensation and motion, the movements of respiration being insensible. All these symptoms may by encreasing terminate in those of asphyxia.

*Effects produced on the Fœtus by the Diseases of the first Stages of Pregnancy.*

If the embryo, when it is formed, or when the ovulum is fœcundated, is produced from depraved germs, it participates in their defects, and contains within it a source of deleterious principles, whose development may be moderated by physical education, but cannot be stopped. Hereditary diseases may, if they are recent, be cured by the resources of art; when inveterate they will be cured only by nature's efforts, that is by time, after passing through several generations.

All the diseases of the mother during the period of pregnancy produce on the child the same final result: they all prevent its regular development; but they produce this effect by acting on the fœtus, some directly by compression, others as it were negatively, by depriving it of the materials necessary for its development.

Without seeking to penetrate into the difficulties of *pathological physiology*, let us examine the effects produced on the child by diseases of the mother during pregnancy. During the efforts of vomiting and coughing, the muscles of the abdomen are strongly contracted; the uterus becomes compressed, and the fœtus may suffer from it. Cardialgia, occasioning disturbance in the functions of digestion, affects all the nervous system, and keeps the fœtus in a state of painful constraint. The same may be said of the colics occasioned by delicacy and irritability, and which are seated chiefly in the uterus. When the mother experiences spasmodic pains, general or partial, occasioned most frequently by mere surprize or sudden fear, it is not to be supposed that the infant can be tranquil under such circumstances. By being developed in the turmoil of the functions of which it must be the product, the child will retain the imprint of this confusion; it will be born weak, and with a bad constitution. The shocks which violent hiccup communicates to the uterus by the contraction of the abdominal muscles compress and torment the fœtus. In tenesmus the viscera contained in the pelvis are made to contract with pain. The uterus compressed by the muscles of the pelvis contracts on the fœtus, and interferes with the delicate progress of its development. Such compressions, being frequently renewed, keep up in the parts which are the seat of them, and especially in the uterus, a constantly increasing irritation, which threatens the fœtus with all the dangers of inflammation. Fever is lighted up, the child suffers more and more, and soon dies. Dizziness has no influence on the fœtus except through the causes which produce it; it then becomes exposed to all the consequences of organic and functional disturbances of digestion, and thus dizziness may be ranked among the affections which deprive the child of nourishment.

Distaste for food, inordinate and depraved appetite, by exposing the mother to the inevitable consequences of bad nourishment, impair the purity of the liquids with which the fœtus should be nourished, and on which the strength or weakness of its constitution will depend. Intestinal discharges, by disturbing the process of digestion, deprive the liquids and solids of their reparatory elements, and the blood becomes impoverished. Hence, with respect to the fœtus, there are the same privations, as those which occur to the infant at the breast when its nurse is insufficiently or badly nourished.

Periodical discharges of blood in pregnant women with a strong constitution, when they are not too heavy, have the effect of preserving the mother and fœtus from certain effects of plethora, which would impede the nutrition of the child. The same, however, must not be said of those discharges called hæmorrhages, and which do not come like the preceding from vessels destined to yield the blood of the menses, but from the interior of the uterus, whose vessels have become relaxed. These losses of blood, like the intestinal fluxes and the cachectic state of the mother, rob the fœtus of the nutricious juice necessary for its growth. The element of nutrition for the fœtus becomes insufficient every time the impoverished blood of its mother which was to yield it, does not find the means of repairing its losses in a sufficiently rich chyle.

Thus all the untoward circumstances which befal the mother re-act upon the child and arrest its development. If the child does not perish in utero, and if it should see the day, it is only to live a sickly life, from which it will probably never emerge. The principles of debility and of disease, which it has received, whether from the germs of its conception, or from the elements which should keep up uterine life in it, are so many sources of chronic diseases. Hence delicate, feeble children, and calamities without end for society, as well as for private families. We shall endeavour to point out the means of preventing all this.

The maternal feeling is not one of those feelings which it is necessary to endeavour to excite in order to obtain from them all the good of which they are capable; but by its tendency to go into excess, and by the multiplicity of circumstances which call it into action, it is one of those feelings which require to be instructed and directed. To be sure a mother's solicitude has the advantage of instinctive inspirations more valuable than any precepts of ours ; but we know full well that ignorance and prejudices often blind it, and carry it beyond the natural indications.

Hiccup arises most frequently, in pregnant women, from bad digestion, and from the presence of undigested matters in the stomach. It may result also from congestion in this organ. It is in this way that the impression made on the diaphragm by the pressure of the stomach on it, manifests itself. Hiccup is more or less frequent according to the degree of the irritation which excites it. In acute diseases inflammation may cause it; it may come on as the effect of surprise or sudden fear. Pregnant women being already predisposed, by their great susceptibility of impressions, to surprise, and fear, they will easily see of what importance it is to keep their minds free from all such occurrences, from those prejudices which seldom fail to disturb young mothers either for themselves, or for their child, more especially during their first pregnancy.

Intestinal discharges comes on in pregnant women often in consequence of excessive indulgence in food, and more especially if it be unwholesome. Such matters disturb the great function of the alimentary canal, which sometimes expels them rapidly, and sometimes retains them for an indefinite period. Hence arise a deficiency of nutrition and the disastrous consequences of an irritation which continues after the discharge of these unassimilable substances, by the depravation they cause in the digestive juices, and by the organic modifications which they leave after them. Women must be careful to resist those longings for unwholesome food which are never so imperious as that they may not be overcome. Those women would be culpable both with respect to themselves and to their child who, when apprized of the mischievous consequences of indulging in such abuses, would still entertain the foolish idea of wishing to render themselves interesting and to draw attention to themselves by such indulgence.

Excremental diarrhœa is usually the effect of an immoderate use of crude, unwholesome food; of the abuse of heating drinks, and of excessive indulgence in food even of a good quality. These indigested substances remain some time in the intestine, become corrupted there, irritate it, and produce diarrhœa, which generally is not of long duration, especially when, for the purpose of checking it, a proper regimen is adopted; but if the patient neglect to pay scrupulous attention to the nature of its causes, the evil continues its progress. Serous diarrhœa arises from a series of laborious digestions. The glands, whose office it is to supply the intestines with the viscid matter destined to line their parietes in order to facilitate the passage of the excrementitious matters separated from the chyle, now retain this matter, which becomes more dense and acrid, and irritates the mucous membranes, and thus is produced serous diarrhœa. If the digestive powers are not re-established, this diarrhœa becomes chronic and colliquative. Bilious diarrhœa arises, in consequence of bad digestion, from the depravation of the bile, which produces on the membranes of the intestinal canal the effect of a drastic purgative.

Dysentery has also for its anatomical cause certain consequences arising from irregularities in diet. Two causes may occasion the flow of the menses notwithstanding pregnancy: plethora, and relaxation of the vessels. In plethora it is the discharge of a superabundant blood beneficial to the mother and child, provided it be not excessive. When natural, it is periodical and occasions no disturbance in the functions nor any pain. When it is not natural, as in delicate, lymphatic females, it takes place at the expense of the strength of the mother and of the nutrition of the child. In this case, as we have already mentioned, it is a loss distinguished from the regular menstrual discharge by its variable duration, its irregular periods, and by the absence of the signs of plethora. The slightest exertion renews it. The patients become faint and languid: they experience a sense of shivering, momentary horripilations, nausea, anxiety, a sense of stifling, and palpitations; lumbar pains towards the pubis, spasms in the hypogastric region, or rather in the organs of gestation. If these consequences continue long, they endanger both mother and child. Among the causes of these losses of blood, we should principally note previous miscarriages, fluor albus, excesses at table, more especially an indulgence in spirituous liquors, frequent watchings, violent passions of the mind, &c. Serious losses of blood may arise from some accident which has happened to the fœtus, and which has acted on the adhesions of its covering to the uterus, and thus separated the vessels which united the uterus to the placenta.

The separation of these two organs is to be prevented by avoiding the causes which may induce it, viz. falls, violent efforts, convulsive coughs, sneezing followed by a great shock, attacks of hysteria, &c.

It is after the disturbances induced by bad digestion that the cachectic state, so fatal to the infant, is generally established in the mother.

We shall close our examination of the causes of the diseases of the first period of pregnancy by that of the causes of syncope and swooning. These depend on the impoverished state of the liquids, and on relaxation of the solid parts of the system, induced by sanguineous discharges; on the slightest motion, or the least mental excitement, women in this state are liable to a series of annoyances, their limbs bend under them, and they fall into an alarming degree of inertness. It is by removing the debility that these consequences are to be obviated.

*Influence of Air.*—Though the fœtus does not breathe in utero, still it is influenced by the air; as a vitiated atmosphere alters its constituents just as well as if it respired. If the south wind prevails during the Winter, if the season is rainy and the Spring cold, pregnant women, who are to be confined in this latter season, are very much exposed to miscarry; if they go their full time, their children are languid and weak. Wherever the South wind prevails during Winter, it keeps the atmosphere more rarefied, consequently lighter, and not heavier, as is usually stated; this renders the respiration more difficult, since the lung receives a less quantity of air. At the same time that the air is rarefied by the South wind, it is hotter; and if humidity be united to this heat, the solids of the system become relaxed; the density of the fluids diminishes, and the diseases depending on this state of the system manifest themselves. Under such a combination of circumstances miscarriages must be frequent, and the children that may be born at the full term cannot but be miserably feeble; and if after such a Winter a cold Spring should come on, few children can hold out.

All derangements of the seasons are injurious to pregnant women. Consequently every season that is too hot, too cold, too moist or too dry, exercises on the fœtus a mischievous influence, which disturbs its functions and enfeebles it, if it does not destroy it. All these derangements and irregularities of the atmospherical constitution merit especial attention on the part of pregnant women, in order that, by adopting the necessary precautions, they may secure themselves against the diseases which may befal them and their child. The variations of the atmosphere and the sudden changes of the seasons produce in the system, both of mother and child, corresponding changes, equally sudden and unexpected; they surprize the body before it is yet prepared to encounter on equal terms the external agents which assail it.

At all periods of pregnancy, but chiefly at the first period, the infant will not possess within itself resources sufficient to resist such attacks. From all this it appears how essentially necessary it is that pregnant women, especially those of a delicate constitution, should be able to appreciate and select the air which is most suitable to their temperament, and to the nature of the inconveniences to which the character of their constitution exposes them. Provided they reflect carefully, they will be better able to select for themselves than the most skilful physician could do for them, the atmospheric medium best suited to the peculiar infirmity of their constitution. If of a dry temperament, a hard, inelastic organization, they will select a moist atmosphere, which will have a tendency to impart suppleness to the tissues. They will find themselves ill at ease if exposed to a keen air, or to the North wind. They will avoid very elevated situations, the sea-coast, &c. If of a humid temperament, and a soft organization, they will prefer a keen dry air, in which they will always find themselves sustained and strengthened. They will avoid moist and marshy grounds, low situations, the vicinity of rivers, &c. If they cannot choose the atmosphere most congenial to them, they should be careful to prevent, by all possible precautions, the ill effects of that in which they are obliged to live.

It must be admitted that the precepts which have been here laid down, are not suitable to all pregnant women. The conditions of life would require a special set of hygienic rules for each ; so that to draw up a truly practical course of hygiene, it would be necessary to enter into the detail of all the cases that may possibly occur in the different conditions of life. This however being scarcely possible, we must be content to lay down general directions and rules. We must first suppose that women have not to devote themselves to any occupation capable of diverting their attention from what they owe themselves during pregnancy. To those who are so circumstanced, the precautions about to be given may at least prove useful. With respect to those who by the exigencies of their circumstances, cannot profit altogether by our counsels, if they do not find in them every thing suitable to them, they will learn from them at least to withdraw themselves from the most injurious influences. They will know what is beneficial and what is injurious to them.

When excessive heat prevails, they will naturally select during the day the coolest place, just so far as to keep the heat of their body moderate. They will keep their rooms cool, say some authors, by strewing leaves and flowers over them. Such a practice, useful as it may be during the day, would be mischievous at night. In the light of the day, in fact, the green parts of the plants absorb carbonic acid from the air, decompose it into carbon which they absorb, and into oxygen which they give out to the atmosphere so as to keep it pure. During the night, on the contrary, they absorb the oxgen of the air, which thus becomes less respirable, and exhale carbonic acid, which renders the atmospheric air impure. From these data we may explain what would happen to a pregnant woman, who should scatter flowers over her apartment, into the interior of which she would intercept the entrance of the light, in order to keep out the heat, so as to keep up in it a kind of half-day. Under such circumstances the pregnant woman, whose functions are performed with enough of difficulty in consequence of her state, would become a prey to a sort of slow asphyxia, ruinous both to the mother and the child. It is in consequence of the great quantity of carbonic acid given off in crowded rooms and large assemblages of persons, that pregnant women are advised to absent themselves during their pregnancy from such places, in which they cannot admit from without a quantity of pure air capable of counterpoising the impure air exhaled by the persons so assembled.

They will therefore be careful to keep the places where they live cool and healthy by allowing a circulation of air, and by keeping the windows shut to the South and open to the North. To moderate the heat which they may feel within them, they will have recourse to the use of cooling vegetable infusions, such as light lemonade, &c. In the use of such drinks they should consult their stomach. If they complain of sourness of stomach, acids are not fit for them, and the use of syrups should be interdicted, as the sweetest syrups often become sour. In such cases water in which some nitre has been dissolved is the best drink.

During Winter they must secure themselves from the impression of cold. Their good sense will not yield to the caprices of fashion, and they will not expose their chest uncovered to the mischievous effects sure to result from such a compliance with usage. If at all times the insensible transpiration is useful to women, it is particularly so during the period of pregnancy. The excrementitious, transpirable matter, retained in the system becomes mixed with the nutritious juices of the fœtus, and communicates its pernicious qualities to it. A moderate degree of heat must be kept up in their dwelling. Too great a heat rarefies the internal air. Hence for the mother disturbance in the functions of respiration and circulation; uneasiness, and sleeplessness, which, with respect to the fœtus, prove so many causes of death.

*Regimen of Pregnant Women.*—To establish constant rules with respect to the diet of pregnant women, it would be necessary to consider in all their infinite variety, the various contingencies which complicate, or may complicate the state of pregnancy; that is, we must foresee and suppose all possible cases. We shall confine ourselves to include them under some general rules, leaving the physician to follow, in a great variety of cases that may occur, the indications which may present themselves at the time.

The diet of pregnant women should vary according to their strength, constitution, habits, according to the nature of their annoyances, and according to the periods of their pregnancy, &c. &c.

Aliments injurious in their nature are principally so to pregnant women: such are indigested, heating, irritating and diuretic aliments; those which develope much gas, salt, smoked, spiced meats, &c.

The disturbance of the functions being progressive, the system becomes gradually prepared for the progressively increasing modifications which it undergoes, and the health of most women, during their pregnancy, does not deviate so far from its normal state, as to induce the necessity of any serious change in the manner of living.

Robust women, accustomed to exercise and exertion, should continue the use of strong food, provided it be wholesome; those who are delicate, should use succulent food, always choosing that most easy of digestion. If in some cases there be a necessity for changing the mode of living of pregnant women, it would be dangerous to do it abruptly. If the woman used, before pregnancy, unwholesome diet, and if, on becoming pregnant, she wished to take proper nourishment, suitable to her situation, she should do so with considerable circumspection. We should also gradually prepare the resistance to be made to depraved longings, so as not to thwart them abruptly.

Those who feel distastes, nausea, and a sense of fulness, ought to subject themselves to a moderate diet, so as not to interfere injuriously with the fœtus; they should not restrict themselves to the use of aliments for which they feel a distaste: in this case, Nature herself often points out the best kind of food: we should observe the preferences with which she instinctively inspires women, and should make it a rule to follow these natural indications. If she feels a dislike for animal food, she should choose among vegetables, not those which may be the best, but such as she may prefer, of whatever quality they may be. We do not mean to say that we should yield to all the caprices of a pregnant woman, but only that we must not resist those cravings which evidently are not injurious.

The physician should carefully investigate the causes of the distastes, and of the irregular appetite. Should they arise from an alteration of the digestive juices, he should do every thing to bring back these fluids to their natural state. If they arise from the presence of slimy matters in the primæ viæ, he should endeavour to remove them by gentle laxatives. In the first period of pregnancy, irritating purgatives would injure the fœtus and might even destroy it. If there be some critical circumstances, in which we may be obliged to have recourse to them, it is for the physician alone to decide with regard to the indications taken from the disease, its symptoms, causes and dangers.

To remove *acidities* of the stomach alkalies are to be employed. If such symptoms are occasioned by a change which has supervened in the state of the solids, for instance, by the tone of the fibres being increased by irritation, or by their becoming relaxed; in the former cause diluents and demulcents are to be employed; and, in the latter, means of an entirely opposite character. In case of considerable relaxation of the solids, it used to be recommended to season the food with mild aromatics, but in small quantity. For this purpose canella was prescribed.

Should the cause of these modifications of the digestive functions be the mere displacement of the organs, we should endeavour to place the woman in such positions as may be most likely artificially to restore the equilibrium. Whatever be the distate for food felt by a pregnant woman, she must not restrict herself to too severe a regimen. The fluids of the system, when they are not repaired and renewed by sufficient nourishment, undergo chemical changes; they soon become depraved and cause disturbance in the several functions.

Neither pregnant women, fœtuses, nor children can tolerate abstinence; it is injurious to them even in their diseases, when it is carried too far, and especially when the pregnancy is far advanced. There is required on the part of the physician the most assiduous attention; he has ample material for serious reflexion for the health both of the mother and child, when he is called in to attend a pregnant woman in sickness. We do not here allude to the method of treating the disease medically, the state of pregnancy being taken into the account; it is now a question of hygiene, viz. of the regimen to be recommended in such a case: to reconcile aliment with disease, and its treatment with the annoying sensations experienced by a pregnant woman, to vary it according to the symptoms and the indications which may present themselves for each patient, to take counsel from experience, and instruction from science, such are the duties of the physician under such embarrassing circumstances. Such is all that can be here said on this important subject.

What has been just said on the subject of regimen, is applicable to all the periods of pregnancy; there is, however, a difference to be established in this respect between the commencement and latter periods of pregnancy. When the fœtus has acquired a certain degree of development, the mother must supply it with a corresponding portion of nutritive materials; she should therefore never subject herself to severe or prolonged abstinence. Another remark which we think necessary in this place, is this: the compression which the stomach undergoes towards the end of pregnancy requires that the sufficient quantity of food be taken in divided portions; a little at the time and often. At the commencement of pregnancy, on the contrary, the fœtus requires but little nourishment. Its development takes place imperceptibly; a too abundant nutritious juice would produce dangerous engorgements in its imperfectly developed organs. The fœtus would thus perish from the superabundance of the materials destined to favour its regular evolution. We shall now give some advice with respect to the liquids most suited to pregnant women.

Pregnant women ought, for the sake of their child, to pay great attention to the quantity and quality of their drinks. In the first period of pregnancy, more especially, the fœtus is of an extremely delicate organization; isolated in the uterus, it adheres to it by only a very feeble force. The slightest accident affects it and disturbs the regularity of its nutrition; a mere nothing almost will then compromise its fragile existence. The *embryonic* state of the fœtus, therefore, will not admit, on the part of the mother, of the abuse either of aqueous drinks, or of spirituous liquors. It is when they are taken in excess, that aqueous drinks may injure the child. With respect to strong liquors, they are absolute poisons to the infant, their action being to harden the tissues and coagulate the fluids of the system. Animals furnish us with a striking instance of sobriety as a preventive mean; among them the mothers, during the time of gestation, are exempt from the accidents to which women are exposed; this exemption they owe to their sobriety, to the choice they make of that kind of food which is always suitable to their nature.

The state of pregnancy, whilst it is a physiological and a natural state, is, notwithstanding, to the female the occasion of strange feelings and sensations, which place her, in some measure, in the intermediate place between the state of health and that of disease. Accordingly, in this hygienic sketch, we shall not attempt to treat of therapeutic substances: but as the woman's state may occasionally call for the employment of medicinal substances, it may not be amiss to make some brief and general remarks on the action of medicinal substances, among which pregnant women will recognise those of which they sometimes make an injudicious and dangerous use. When instructed, on the one hand, respecting the different properties of medicines, and, on the other, regarding the organic conditions and morbid dispositions in which the state of pregnancy places the mother and child, they may become more prudent.

With respect to the preparations, the use of which is sometimes recommended to pregnant women, in certain cases, by ignorance and prejudice, we shall here make some remarks. As our limits will not allow us to attack them individually, we shall include them all under one general head of instruction concerning the properties of certain classes of medicines. This instruction being intended to prevent abuses in the employment of medicines and not to lay down rules for prescribing them, we shall refer to those only which are most known; among which are those most frequently employed by people in ordinary life, who will find in this enumeration some notice taken of the noxious qualities of certain substances whose employment has been consecrated by abuse, by introducing them on our tables among our ordinary articles of diet, whether solid or liquid.

1st. Tonic substances: these increase the tone of the organs; their action is slow, but of long duration. They are for the most part bitter; in some bitterness and astringency are combined. Some have an aromatic odour. Tannin, gallic acid, and the bitter principles predominate in simple bitter tonics; as lesser centaury, the hop, carduus benedictus, chicory, &c.

Bitter astringents: cinchona, willow, &c.

Aromatic bitters: absintheum, camomile, &c.

Mineral tonics: iron, and its preparations, constitute a very powerful tonic agent; it is employed in all diseases characterised by paleness, debility and an impoverished state of the blood: iron-filings, chalybeate wine; various pills, whose base is some preparation of iron, as Bland's pills; a preparation of chocolate and iron, which goes by the name of chocolat ferrugineux de Colmet-d' Aage. Some practitioners will have it that there are two methods of tonic medication: the one analeptic, and the other neuro-sthenic. The first includes only iron; the second includes all the *bitters*.

2nd. Astringent substances: they have a more or less acid taste. Their action scarcely occasions any general phenomena. They produce a condensation of the organs, and an occlusion or closing of the minute vessels; they diminish and sometimes suppress natural discharges. Hence their employment in hæmorrhages, mucous discharges, diarrhœa, &c.

Mineral astringents: sulphuric acid; nitric acid (with alcohol); alum, tartrate of iron and potass; acetate of lead; and lime-water.

Vegetable astringents: the bark and acorn of the oak; rhatany; pomegranate; the red rose; agrimony; sorrel; syrup of quinces; dog-rose; walnut; lemon.

3. Stimulating substances: they almost all have a strong, penetrating, aromatic odour, a hot, smart taste; which, however, is not of any duration. They quicken the circulation, increase the animal heat, and seem to exert a specific action on the nervous system. The most energetic among them have an immediate action; these are what are called *diffusibles*, such as alcohol and ethers. Some exercise their action on almost all parts of the system, whilst others at the same time stimulate more particularly one organ or function; ergot of rye, rue, savine, direct their action to the uterus; Virginian snake-root to the bronchial mucous membrane, &c.

Vegetable stimulants: among these may be classed the various generous wines; alcohol, and the ethers; the various essential oils; camphor, musk: ginger, pepper, orange flowers and leaves, aniseed, valerian, balsams, gum-resins, and others too numerous to mention.

Stimulants from the animal kingdom: ammonia, and its preparations; phosphorus, creosote, musk.

Specific stimulants of the uterus, or substances, which excite the menses and bring on abortion: ergot of rye, rue, savine, saffron, fœtid gum-resins, ammoniacum, ferruginous preparations, &c.

These substances having a specific stimulating action on the uterus, it will be obvious that their employment, during pregnancy, even though they may not be pushed far enough to bring on abortion, may prove fatal to the child; and yet some of these substances are frequently recommended during pregnancy for the use of the debilitated mother and her infant; as, for instance, the preparations of iron. The contradiction is but apparent. In this case, in fact, these substances are not recommended to combat the debility of the uterus, and as local stimulants of this organ, but, in consideration of the general debility, as tonics and general stimulants, they being in fact tonics rather than stimulants.

4th. Debilitating substances: these diminish the action of the circulation, and consequently that of most of the great functions of the animal economy. This class includes two orders of substances.

Cooling substances, or temperants: lemon-juice, tartaric acid, vinegar, acidulated fruits, such as oranges, gooseberries, strawberries, cherries, &c. 2nd. vegetable emollients; gum, marsh-mallow, sago, tapioca, rice, barley, sweet-almonds, chocolate, &c.

Emollients from the animal kingdom: veal broth, gelatine, isinglass, cow', asses', and goats' milk, the flesh of young animals.

The frequent use of diuretic medicines must be avoided by a woman in the state of pregnancy, such as squills, foxglove, aconite, colchicum, &c.

There are several of the substances just enumerated, which individually are incapable of injuring; but as they may enter into the diet which some women prescribe to themselves in combination with others of the same class, it was as well to set down as injurious the entire list of these articles, though separately they may be very harmless. Thus, for instance, the diet of a pregnant woman, if exclusively constituted of emollient alimentary substances, may, by depressing the principal functions in the mother, retard the development of the child.

It will be observed that, among debilitants, will be found a considerable number of nutritious and analeptic substances. Such, for instance, are the different fæculæ, gelatine, harts-horn jelly, &c. These substances, in consequence of their ready assimilation, may constitute a nutritious diet for an enfeebled constitution, which, during convalescence from a disease, requires to recover its strength gradually; but the exclusive use of such aliment would constitute a debilitating regimen for a pregnant woman; who, without having lost any of her strength, requires to augment it, in order to share it with the new being, which is being developed within her. There is, however, a period of pregnancy, that of the early months, when the woman must use light food and that of easy digestion, such as fæcula, animal jellies, &c.

After having alluded to the action of medicines on the system of the mother and child, we may observe that iced drinks, whatever be their constitution, cause violent colics and may bring on miscarriage. We shall now make a few remarks on heating drinks and on food of the same quality.

The stimulating action of substances with a strong, penetrating, aromatic odour and with a hot taste, is intense and immediate. The continued use of such meat and drink hurries the circulation, increases the animal heat, agitates the whole nervous system, and thereby disturbs the several functions; pains are felt in the region of the heart, hæmorrhages follow; and if the fœtus, the victim of these abuses, does not perish, it will carry, from its birth, the germs of constitutional diseases, such as may manifest themselves in a frame prematurely vitiated at the very fountain-head of life. From this it must not be inferred that we are to proscribe from the regimen of a pregnant woman those cordial drinks so often necessary in consequence of the faintings and swoonings to which the pregnant state exposes them, We should even endeavour to prevent their occurrence by the use of generous wine. If the woman is of a delicate organization, or is afraid of giving birth to children that are liable to inherit the feeble constitution of their father, she should make use of tonics during the entire period of her pregnancy. Among the substances endowed with a tonic property, there is none whose powers are better established, or more universally recognised, than iron and its various preparations. A happy application has been made of this substance by M. Colmet-d' Aage, an apothecary in Paris, when he conceived the combination of a powerful ferruginous preparation with an article of ordinary diet so agreeable to the taste as chocolate, so as to produce a medicated aliment easily borne by all stomachs. However it happens that in ferruginous chocolate, the mixture of iron and chocolate retains no trace of the inky and styptic taste of iron, it is a privilege which this preparation enjoys exclusively; it is also that which is most easily given to infants, and for the longest time to adults. This preparation is particularly recommended by the first medical men in Paris to pregnant women, whose capricious stomachs perform their functions so badly, and whose appetite is so often perverted and destroyed.

